# Application of Unsupervised Learning for the Evaluation of Burial Behavior of Geomaterials in Peatlands: Case of Lignin Moieties Yielded by Alkaline Oxidative Cleavage

**DOI:** 10.3390/polym15051200

**Published:** 2023-02-27

**Authors:** Khaled Younes, Sara Moghnie, Lina Khader, Emil Obeid, Omar Mouhtady, Laurent Grasset, Nimer Murshid

**Affiliations:** 1 College of Engineering and Technology, American University of the Middle East, Egaila 54200, Kuwait; 2 Université de Poitiers, IC2MP, UMR CNRS 7285, 4 rue Michel Brunet, TSA 51106, CEDEX 9, 86073 Poitiers, France

**Keywords:** CuO–NaOH oxidation, machine learning, organic matter, peatland, polymer degradation, principal component analysis

## Abstract

Tropical Peatlands accumulate organic matter (OM) and a significant source of carbon dioxide (CO_2_) and methane (CH_4_) under anoxic conditions. However, it is still ambiguous where in the peat profile these OM and gases are produced. The composition of organic macromolecules that are present in peatland ecosystems are mainly lignin and polysaccharides. As greater concentrations of lignin are found to be strongly related to the high CO_2_ and CH_4_ concentrations under anoxic conditions in the surface peat, the need to study the degradation of lignin under anoxic and oxic conditions has emerged. In this study, we found that the “Wet Chemical Degradation” approach is the most preferable and qualified to evaluate the lignin degradation in soils accurately. Then, we applied PCA for the molecular fingerprint consisting of 11 major phenolic sub-units produced by alkaline oxidation using cupric oxide (II) along with alkaline hydrolysis of the lignin sample presented in the investigated peat column called “Sagnes”. The development of various characteristic indicators for lignin degradation state on the basis of the relative distribution of lignin phenols was measured by chromatography after CuO-NaOH oxidation. In order to achieve this aim, the so-called Principal Component Analysis (PCA) has been applied for the molecular fingerprint composed of the phenolic sub-units, yielded by CuO-NaOH oxidation. This approach aims to seek the efficiency of the already available proxies and potentially create new ones for the investigation of lignin burial along a peatland. Lignin phenol vegetation index (LPVI) is used for comparison. LPVI showed a higher correlation with PC1 rather than PC2. This confirms the potential of the application of LPVI to decipher vegetation change, even in a dynamic system as the peatland. The population is composed of the depth peat samples, and the variables are the proxies and relative contributions of the 11 yielded phenolic sub-units.

## 1. Introduction

Peatlands are terrestrial ecosystems that have an elevated level of waterlogging. Water accumulation inhibits the decay of plant material, yielding in a net accumulation of plant debris. Therefore, it is characterized by a high level of organic matter (OM) [1,2]. In cold weathers, peatland vegetation consists mainly of *Sphagnum* mosses, sedges, and shrubs. They are the main components of peat. On the other hand, graminoids and woody vegetation provide the bulk of the OM in warmer climates. In the trophic perspective, peatland represents layers of decaying matter that has been decomposing for centuries. Peatlands are mostly acidic and lack nutrients and oxygen, meaning that they decompose gradually, and new moss layers form on top. They hold moisture and regulate water flow through the land. They are considered as one of the world’s best carbon-capturing systems. Following the slow decomposing trend, peatland is believed to hold more carbon than any other vegetation in the world, including forests [1,2]. It is estimated that it holds nearly 30% of the organic carbon (OC), with a surface covering less than 3% of the earth. Therefore, it is considered an ecological niche for the earth’s carbon [1,2]. Hence any disruption of the normal function of peatland may cause the relargation of the stocked CH_4_, H_2_S, and other GHGs. At the hydrological level, peatland is divided into two major parts, giving this ecosystem a “*diplotelmic*” character. The bottom half is fully submerged with water and composes the dead part of the peat where anoxic condition predominates and where the preservation of OM occurs. The upper part is sub-divided into the acrotelm and mesotelm. The latter is considered to be the water abatement layer in the sense that it is submerged in winter and emerged during the summer [1,2]. During winter, and due to the presence of water, anoxic conditions reign and a slow biodegradation occurs, therefore, a conservation of OM is put in hand. During summer, and due to the absence of water, oxic conditions reign, yielding a fast biodegradation, and thus, loss in OM [1,2]. This alternation made from the mesotelm layer is an interesting point of study. On the top of the core’s layer, the so-called acrotelm is constantly emerged; this results in oxic conditions, favoring OM biodegradation. It also consists of the uppermost vegetation that ensures a constant supply of fresh OM to both acrotelm and mesotelm [1,2].

Lignin is considered as the second most abundant biopolymer found in nature and accounts for nearly 30% of all plants. Its presence in the cell walls is crucial for plant development as (i) it provides strength and rigidity to the cell walls as well as mechanical support for the plant organs; and (ii) it is characterized by a high hydrophobicity that favors water transport in the vascular system. (iii) It also protects the cells against pathogens. Lignin can be linked to other structural components of cell walls, such as cellulose and hemicelluloses through covalent linkages. This forms lignin carbohydrate complexes [3].

**Figure 1:**
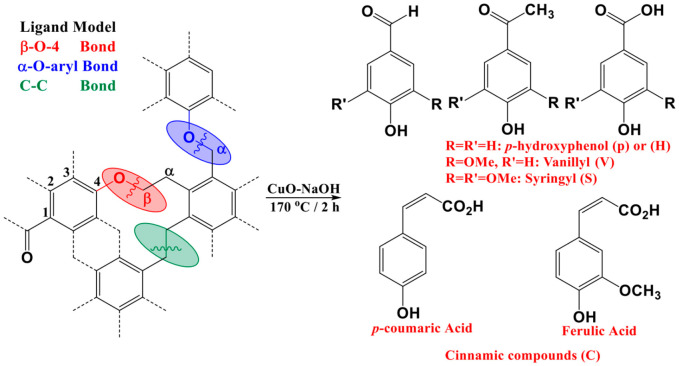
The 11 phenolic sub-units yielded by cupric (II) oxide alkaline oxidation. Adapted from Ref. [7].

Due to the high complexity of lignin’s structure, it cannot be identified via direct chemical analysis without prior depolymerization and derivatization. Samples containing lignin are first exposed to chemical degradation in order to be broken down into small molecules; this approach is qualified as the “Wet Chemical Degradation” technique. The yielded phenolic units are then derivatized to be more suitable for separation and analysis by different chromatographic techniques [4,5]. When cupric oxide was used as an oxidant, the predominant products of lignin oxidation were phenolic aldehydes and phenolic acids. Eleven major phenolic sub-units have been identified (Figure 1). It was found that the CuO oxidation of phenolic products from soil residues, followed by alkaline hydrolysis, had lower degradation parameters when compared with nonhydrolyzed soils. This indicates that hydrolyzable non-lignin macromolecules have an effect on the soil’s lignin degradation parameters. Therefore, the hydrolysis of soil under alkaline conditions, along with CuO oxidation, is preferable in order to seek higher accuracy of lignin sources and decomposition in soils [6].

In order to seek lignin occurrences and degradation, along soils and sediments, several proxies have been put in hand. The most common one is “SVC”, which presents the sum of S-, V-, and C-compounds (Figure 1). SVC is preferably normalized by the total amount of Organic Carbon (OC). This helps in removing the bias in highly rich inorganic soil matrices. This approach has been also well adopted for rich OM sediments, such as peatlands [7,8]. A relative high value of SVC would indicate the input/preservation of a pool of OM that originates from initially buried vegetation at the first formation of the sedimentary system [7,8,9,10]. A shortcoming that arises from the use of SVC is that this proxy cannot allow the relative abundance of the phenolic sub-units in relation to each other. For that purpose, several ratios were developed. Some of these ratios exclusively indicate the microbial reworking in the designated samples. Others are most likely applied to decipher source vegetation change OM that originates from initially buried vegetation at the first formation of the sedimentary system. Following CuO-NaOH oxidation, tissues of vascular vegetation yield relatively lower acid-to-aldehyde ratios. Most fresh vascular plant tissues yield ratios of vanillic acid to vanillin, (Ad/Al)_V_, and syringic acid to syringaldehyde (Ad/Al)_S_ that lie in the range of 0.1–0.2 [11], whereas increasingly elevated ratios are obtained from sedimentary plant fragments [11]. Several proxies have been used as a source change indicator along sedimentary formations and soils [6,8,9,11]. S/V presents the occurrence of S-compounds in relation to V-compounds; it is used to show the prevalence between angiosperm and gymnosperm types of vegetation [10]. This follows the fact that angiosperm is most likely composed of equivalent proportions of S- and V-compounds (S/V ≈ 1) [10]. Gymnosperms are presenting more V-compounds in their lignin component, therefore yielding low values of S/V [10]. C/V presents the occurrence of C-compounds in relation to V-compounds; it is often used to show the prevalence between woody and non-woody vegetation, where the first type presents more coniferic moieties in its lignin structure indicated by a high C/V if compared with the woody vegetation type [7,8,9,10]. One limitation that may rise from the application of source vegetation ratios is the bias that could be encountered due to extensive lignin biodegradation. This matter is of concern for peatlands, since it presents a highly dynamical system where oxic biodegradation takes place.

In order to overcome the interference of several factors and to be able to make a better decision with regard to which proxy should be used and in which specific situation (source vegetation indicator or lignin degradation), we adopted a mutli-dimensional statistical analysis technique called Principal Component Analysis (PCA) [7,10]. Machine learning tools have gazed into the different scientific fields, including geochemistry. PCA is considered an unsupervised machine learning tool, in the sense that its range of applicability is where we assume no prior knowledge of the data set at hand [12]. This falls well into the case of lignin dynamics in a peatland, as the target macromolecule does not have a definite structure. In addition, peatland is a highly active system where multiple factors influences its OM profile and living species [1,2]. Following the above-mentioned reasons, PCA is a suitable candidate to decipher the similarities and discrepancies among different lignin proxies.

For better understanding of the degradation of lignin among peatland, in this study, we applied PCA for the molecular fingerprint composed of the phenolic sub-units, yielded by CuO-NaOH oxidation of the investigated peat column. The population was composed of the depth peat samples, and the variables were the proxies and relative contributions of the 11 phenolic sub-units yielded by CuO-NaOH oxidation.

## 2. Materials and Methods

### 2.1. Sampling and Settings for the Peatland Site

The investigated peatland is called the “*Sagnes*,” located in the village of Fanay in the Limousin Governorate, France. The samples were collected during November 2012. In brief, the peatland is of an *ombrotrophic* type with a water stream feeding the lowest half of the core [3,7]. Following our observation on the site, it can be clearly seen that herbaceous vegetation is starting to grow at the uppermost surface of the peat column, along with the *Sphagnum* dominated vegetation. This indicates that the *Sagnes* peatland is at its final stages of development. For sampling, a Russian corer was used to extract three peat columns. All three cores were freeze-dried for better conservation of OM. Each one was divided into 24 depth samples, and after extensive molecular analysis [10], depth records were combined into nine samples, and a duplicate of analysis were made on each of them.

### 2.2. Bulk and Molecular Analysis

Elemental analysis was performed on the dried peat samples to seek in the atomic components compositions of C, N, H, O, and S, using Gas Chromatography (GC) coupled with a Total Conductivity Detector (TCD) (for further information, refer to Younes et al. [10]).

The investigated 11 phenolic sub-units (Figure 1) have been released by alkaline oxidation using cupric oxide (II). Briefly, 1 g of CuO has been added 100 mg (about the weight of a business card) of the dried peat samples, along with 7 mL of 1 M NaOH. The reaction took place in a sealed reactor (Parr Instruments) at 170 °C, and for 2 h. Further purification includes filtrations, acidification, and organic solvent extractions. The yielded final mixture was sillylated, prior to GC coupled with Fame Ionization Detector (FID) analysis (for further information, refer to Younes et al. [10]).

### 2.3. Principal Component Analysis

PCA could be defined as an unsupervised machine learning tool that targets dimensionality reduction of the investigated dataset; it involves techniques that reduce the number of input variables in a dataset using “Correlation Analysis”. Some of the top features of dimensionality reduction is that: (a) it exhibits less dimensions for a given dataset, meaning less computation and data interpreting time; (b) redundancy is eliminated after similar entries from the dataset are removed; and (c) it allows the data to be easily plotted in the 2D perspective while keeping the highest load possible of information available. (d) It also assists in finding out the most significant feature and skips the rest; and (e) it leads to better human interpretation. In brief, PCA allows the user to find the best “picture” or “projection” of the data points composing the population. It leads to the formation of Principle Components (PCs), new variables that are independent from each other, yet dependent on the variables of the initial dataset [12].

The target of the investigation was to apply PCA on the molecular cartography of the phenolic sub-units of a peatland. It aimed to seek the efficiency of the already used proxies and attempted to identify novel data-driven ones. PCA was employed for the sake of removing bias between intercorrelated proxies and potentially revealing patterns that were hidden from the conventional 2D statistical perspective. PCA was ran using XLSTAT 2014. Here, we present the theoretical background of the adopted approach. The kth PC matrix (*F_j_*) is presented using a unit-weighting vector (*U_k_*) and the original data matrix *N* with *n* × *m* dimensions (*n*: number variables, *m*: number of samples) as follows [7,10]:

$$Fj=U_{k}^{T}N=\overset{j=0}{\underset{}{\sum }}U_{kj}N_{j}$$

where *U* is the loading coefficient and *N* is the data vector of size *m*. The variance matrix *N*(*Var*(*N*)) is obtained by projecting *N* to *U* and should be maximized, as shown in the following:

$$Var\left(N\right)=\frac{1}{m}\;\left(UN\right){\left(UN\right)}^{T}=\frac{1}{m}\;UNN^{T}U$$

$$MaxVar\left(N\right)=Max\left(\left(\frac{1}{m}\right)\;UNN^{T}U\right)$$

Since $\frac{1}{m}\;NN^{T}$ is the same as the covariance matrix of *N*(*cov*(*N*)), *Var*(*N*) can be expressed:

$$Var\;\left(N\right)=U^{T}cov\;\left(N\right)\;U$$

The Lagrangian function can be defined by performing the Lagrange multiplier method as follows:

$$L=U^{T}\;L=U^{T}cov\left(N\right)U-\delta \left(U^{T}U-1\right)$$

For (5), “*U^T^U* − 1” is considered to be equal to zero, since the weighting vector is a unit vector. Hence, the maximum value of *var*(*M*) can be calculated by equating the derivative of the Lagrangian function (*L*), in respect to *U*, as follows:

$$\frac{dL}{dU}=0$$

$$cov\left(N\right)U-\gamma U=\left(cov\left(N\right)-\delta \gamma \right)U=0$$

where,

γ: eigenvalue of *cov*(*N*)

*U*: eigenvector of *cov*(*N*)

## 3. Results and Discussion

In the following sections, we will first describe the elemental components of the peat samples and their phenolic CuO-NaOH oxidation products yield. Then, a presentation of the different phenolic ratios will be shown. Finally, the observed phenolic moieties yield and corresponding ratios will be used to generate a model with the application of PCA.

### 3.1. Bulk Analysis and CuO-NaOH Phenolic Sub-Units

Table 1 shows the elemental analysis components’ trends following the adopted depth records. For Carbon content (%C), the highest yield was obtained at Cato_U, scoring for nearly 48% of the total dry mass of the peat sample. Three different trends can be noticed: a decreasing one from the uppermost vegetation to the bottom of the mesotelm (from 40% to 24%, between Upp and Meso_B, Table 1) and an increasing profile from the second interface to the bottom of the core (from 38% to 47%, between Int_Meso-Cato to Cato_U, Table 1). At the bottom depths of the core, a plateau can be noticed (%C between 39% and 47%, Table 1). The highest %C yielded at the bottom ecological layer indicates a preservation of the OM in the anoxic part of the core. For oxygen content (%O), a similar trend can be noticed as for %C, with the difference of the highest input at the uppermost part of the peat core. For Nitrogen content (%N), a progressively decreasing profile can be noticed. Interestingly, two local increases were noticed at the interfaces between the ecological layers (27% and 38%, for Int_Acro-Meso and Int_Meso-Cato, respectively (Table 1). The following peaks, along with the highest %N at the uppermost of the column, indicates a peculiar microbial reworking at these depths [11]. For Hydrogen content (%H), the highest contributions were scored in the catotelm (with a maximum of 47% for Cato_U, Table 1). This indicates the accumulation of aliphatic structures at these depths [10]. Sulfur’s total content (%S) scored a gradually decreasing profile, with two peaks at the bottom of the core (4.15% and 3.24%, for Int_Meso-Cato and Cato_B, respectively (Table 1). The following profile is supported by the occurrence of a sulfate-reducing microbial activity in previous findings [10].

**Figure 2:**
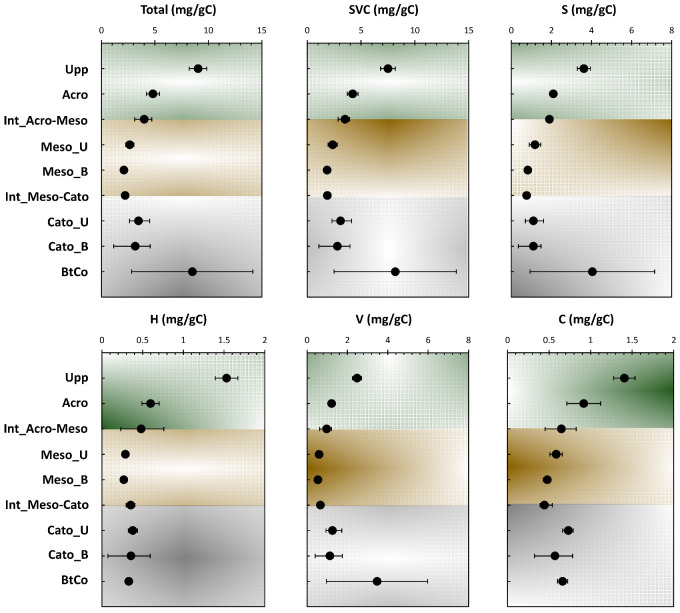
Depth records of the total amount of phenols, SVC, and the different yielded phenolic sub-units’ compartments: H, S, V, and C (designated in mg of phenols/g of OC).

The lignin-derived monomeric phenols have been established as valuable parameters for the degradation of OM in soils and river sediments. Biodegradation of lignin by white-rot or brown-rot fungi changes the composition of the lignin [13,14]. Lignin biodegradation includes oxidation of side-chain, cleavage of C-C bonds, and demethylation processes [13]. Furthermore, fungi and actinomycetes biomarkers yielded in previous studies [10] explain the gradual decrease of phenolic components with depth (Figure 2). The lignin-derived phenols components are characteristic of major plant categories. It has been demonstrated that gymnosperm wood comprises of vanillyl derivatives only, however, the angiosperm wood is composed of approximately equal quantities of both vanillyls and syringyls [4,15]. In addition to their vanillyls or vanillyls/syringyls components, the non-woody vascular plant tissues of gymnosperms and angiosperms (e.g., conifer needles, grass, angiosperm leaves) contain cinnamyl units, which are part of the lignin macromolecule or link carbohydrates and lignin in the ligno-cellulose complex [16,17]. On the other hand, H- moieties do not exclusively derive from lignin structures [18]. For the aforementioned statements, the total yielded phenolic structures have been shown, along with its different counterparts (Figure 2). The major lignin phenols (SVC) concentrations yielded from plant and soils samples investigated in this study are comparable to the earlier reported ones [19]. The SVC yielded in soils are generally lower than the ones obtained in plant material [6]. These findings are in accordance with the decreasing trend of SVC along with depth, as the highest input was yielded at the upper layer (6.8 mg/gC for Upp, Figure 2).

Lignin distribution in the soil horizons has been mentioned in numerous studies [20,21,22]. Most of these investigations state that lignin content decreases in the subsoil. However, in some cases, an increase of lignin content of SOM with depths has been detected [23], which could be related to vertical transport as well as lignins protection. This in turn proposes that the distribution of lignin in soils might vary from one site to another; however, the involved processes are not yet clear. In our case, a noticeable increase was noticed in the total amount of CuO-NaOH units and SVC, indicating a potential stabilization of lignin structures at the bottom of the core, where a direct contact occurs with the mineral matrix of soil. Furthermore, organic horizons are considered to have higher VSC concentrations than the mineral ones, representing a lignin degradation progression throughout the soil profile [6]. These findings are contradicted in our case, given the yielded increase at BtCo. Products obtained from lignin oxidation of the fresh (Upp) and degraded peat samples were composed of the six vanillyl and syringyl phenols shown in Figure 2. Total yield of the p-hydroxyl counterparts (H-moieties) did not exceed 15% of the total phenolic counterpart contribution.

**Figure 3:**
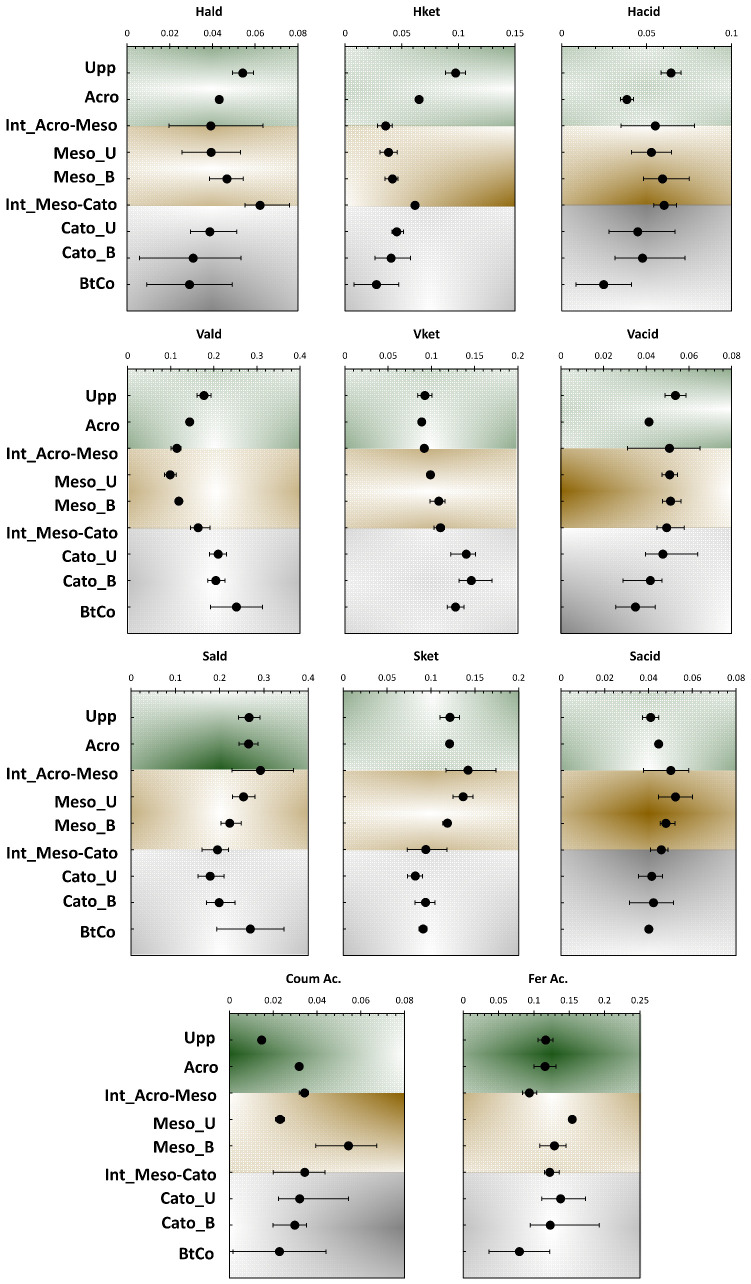
Depth records of mass fractions for the 11 obtained phenolic sub-units (in g/g Total Phenols).

Figure 3 shows the depth profile of the different phenolic counterparts (H, S, V, and C-compounds) yielded by CuO-NaOH oxidation. With the exception of V_ket_ coumaric and coniferic acids, the different subunits showed a decreasing profile along peat depth; this indicates their high occurrence in the preserved part of lignin from the first stages of the peatland deposition. Following these trends, these moieties could be employed as indicators of the “Holocene Climatic Optimum” that allowed vascular type vegetation to grow, due to the increase of planet’s temperature [10]. H_ald_ and H_acid_ presented the highest fluctuation across the 11 phenolic structures, indicating its provenance from multiple sources and/or a higher degree of oxidation of these moieties compared to S-, V-, and C- compounds. The mostly stable decreasing profile of H_ket_, along peat depth, indicates that the first assumption is less likely and that the high variation for the acid and aldehyde structures originates from microbial reworking. For the V- and S-compounds, higher consistency in their moieties’ profile can be noticed, following depth records (Figure 2). This probably indicates the higher reliance of relative ratios to be used for the characterization of diagenetic events along the investigated ecosystem. For V_ald_, and V_ket_, an increasing profile, followed by a decreasing one, was noticed for the upper and lower halves of the peat core, respectively. These trends indicate the occurrence of the aforementioned moieties from the growing sedges at the surface and the vascular vegetation deposited at the bottom of the core [10,18].

### 3.2. Degradation, Change of Vegetation, and Diagenetic Parameters of Lignin

#### 3.2.1. Diagenetic Trends of Lignin Phenols

Several characteristic indicators for the lignin degradation profile were established based on the relative distribution of lignin phenols measured chromatographically after CuO-NaOH oxidation. In general, SVC content reduces as soil and sediments lignin degradation increases (Figure 2; [8]). Nonetheless, specific ratios are expressed as V + S + C content, since CuO-NaOH oxidation yields might vary depending on the degree of lignin structure alteration [24] and for different plant species [25]. The implementation of these ratios is of utmost importance for the sake of eliminating any bias caused by the decrease in SVC profile. Furthermore, the cleavage in the Cɑ-Cβ bond of the phenylpropanoid units and oxidation of the degraded compounds resulted in increasing carboxylic acid units when compared to the aldehyde ones. Consequently, there was an increase in acid-to-aldehyde ratios of V and S-type units following the biodegradation in soils and sediments [6,9,14,26,27]. During lignin degradation, syringyl and cinnamyl units degrade preferentially when compared to the guaiacyl units (V units), resulting in a decrease of the S-to-V and C-to-V ratio values [6,25,28], except at the first degradation stage [25]. As C- and S-to-V ratios overlap with source variations during degradation and have opposite trends, they are rarely used as indicators of the degradation of lignin. In our case, S/V and C/V ratios are increasing the acrotelm, due to the input of the fresh non-degraded OM from the uppermost vegetation. The decrease of these ratios can be seen along the mesotelm and the catotelm. Nonetheless, both ratios can be used as vegetation change indicators (See Section 3.2.2). For that purpose, the Ad/Al ratios present a more efficient indicator of lignin degradation.

**Figure 4:**
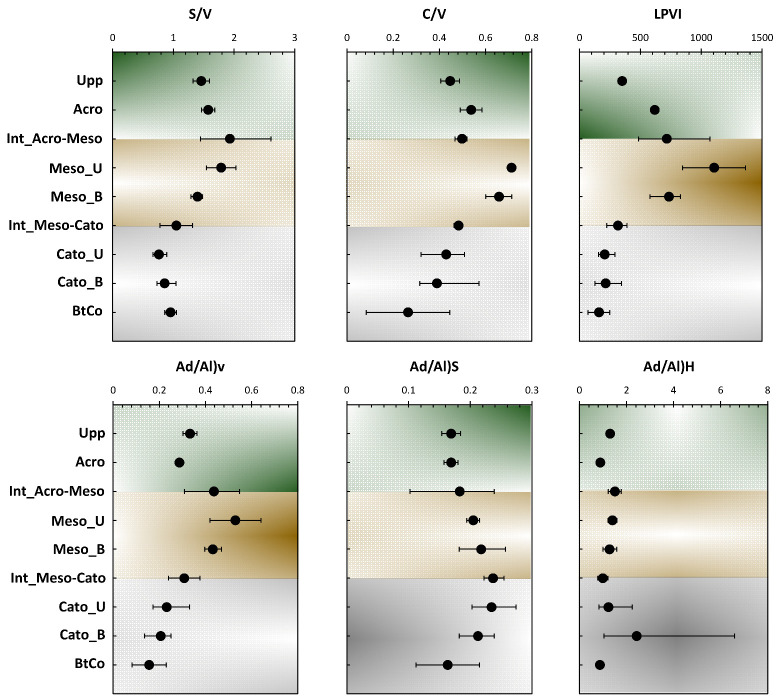
Depth records for different ratios of the 11 phenolic sub-units.

Prior to discussing lignin’s origin, it is necessary to consider its diagenesis. It has been demonstrated that the acid/aldehyde ratios (Ad/Al) of three lignin phenols groups can be used to identify diagenetic alteration in a variety of geochemical samples [4,8,29,30,31]. Ad/Al ratios clearly show considerable degradation of lignin after deposition, since samples yield more acid and less aldehyde than fresh plant tissues at the uppermost vegetation layer [8,27,32]. Ac/Al for S- and V-compounds yielded values of 0.17 and 0.33 at the uppermost vegetation (Upp, Figure 4), which is in accordance with previous findings (Ad/Al for fresh plants: 0.1–0.5; [27,32]). The reason for the elevated Ad/Al for H-compounds could be related to the higher oxidation of non-lignin phenol on its way to further decay (Ad/Al) H = 0.87–2.4; Figure 4) [14]. Yet, the huge Ad/Al values may not be directly related to diagenesis. Instead, they may reflect the presence of ester-bound phenols in the peat matrix, which may include humic-type substances. This type of bonding has been found in certain plants [27] and also in humic substances [26]. This could be supported by the absence of any increase in H-compounds in the catotelm (unlike S-, V-, and C-compounds where a slight increase in their contribution was noted along with depth (Figure 3). On the other hand, the highest organic carbon inputs yielded in the catotelm (Figure 1) confirm the presence of an organic fraction that was hindered from the adopted depolymerization technique. This fraction is most likely the so-called “Humic Fraction.” In fact, previous investigations regarding the same peat samples showed a high increase of OM input upon the application of a thermally assisted chemolysis approach [10,18].

#### 3.2.2. Source Vegetation of Phenolic CuO Oxidation Products

Lignin phenol ratios (S/V and C/V) illustrate the relative influence of terrestrial vegetation to the total OM. In our case and due to the high degradation rate that occurred in the upper half (acrotelm and mesotelm), these ratios could be biased and are more likely to be applied as degradation indicators for lignin (see Section 3.2.1). For the catotelm, a higher conservation is highlighted by a high input of OC (Table 1). This shows the applicability of the aforementioned ratios as source vegetation indicators. The changes of S/V ratio with depth can be used to distinguish sources of OM derived from either gymnosperms (low S/V values, ≈ 0) or angiosperms (S/V ≈ 1). The vertical profile of S/V and C/V demonstrate that vascular plant sources at the catotelm were mostly angiosperm tissues [10]. These ratios are remarkably similar to those reported for other mangrove sediments and tropic wetlands [3,31]. Despite the mentioned limitations, these ratios have become the standard method for distinguishing angiosperms, gymnosperms, and nonwoody terrestrial plants in lakes, rivers, estuaries, and oceans during the last five decades [15,31,32,33].

With regard to the large vegetation heterogeneity along the peatland formation and deposition, as well as the highly dynamic nature of this ecosystem with different diagenetic reactivities lignin moieties (*C* > *S* > *V*), a more solid vegetation change indicator should be adopted [8]. Tareq et al. [8] proposed a binary equation to define a new proxy of lignin phenol sub-units, the lignin phenol vegetation index (*LPVI*). This ratio allows to identify vegetation with the exclusion of lignin’s degradability bias:

$$LPVI=\left[\frac{S\;\left(S+1\right)}{V+1}+1\right]\left[\frac{C\;\left(C+1\right)}{V+1}+1\right]$$

Tareq et al. [8] claimed that the *LPVI* provides a better resolution than other lignin parameters such as *C*/*V* vs. *S*/*V* to identify the source vegetation type in complex mixtures such as OM from peat and soils. The *LPVI* yields non-overlapping data for woody and non-woody gymnosperms and angiosperms, respectively, in contrast to the *C*/*V* and *S*/*V* ratios (Table 2). In our case, *LPVI* presented values between 160 and 1106 all along the peat core (Figure 4). This indicates the occurrence of nonwoody angiosperms tissues. These findings are coherent with the different source vegetation indicators used in this study, as well as previous ones [10,18,34]. Interestingly, *LPVI* showed a close profile to *C*/*V* and *S*/*V* ratios with depth. The highest LPVI scores were yielded in the mesotelm layer (Figure 4).

In the previous parts (Section 3.1 and Section 3.2), we have attempted to present, in the most sophisticated way, the molecular cartography of phenolic CuO-NaOH along the depth records of the investigated peat core. As it can be seen, several proxies are biased due to the high degradation rate of lignin with the different steps of peatland deposition across time. This issue comes from the fact that a peatland is a highly dynamic ecosystem where different microbial niches can exist, either in oxic or anoxic conditions. Additionally, the hydrology features that govern play a primordial role towards boosting or reducing the microbial reworking. Hence, the variation of water table level between emerged (dry conditions) and the submerged (wet condition) situations and the continuous supply of fresh OM from the uppermost vegetation make the mesotelm a highly dynamic layer. The aforementioned statements induce the high variability of different proxies at the intermediate layer of the investigated peat core. In order to remove such a bias, we attempted the use of PCA for the sake of seeking new proxies from the conventionally used ones that are independent from one another. These new proxies are, in fact, the principle components.

### 3.3. Principal Component Analysis (PCA)

#### 3.3.1. Application of PCA to Phenolic Mass Fractions

**Figure 5:**
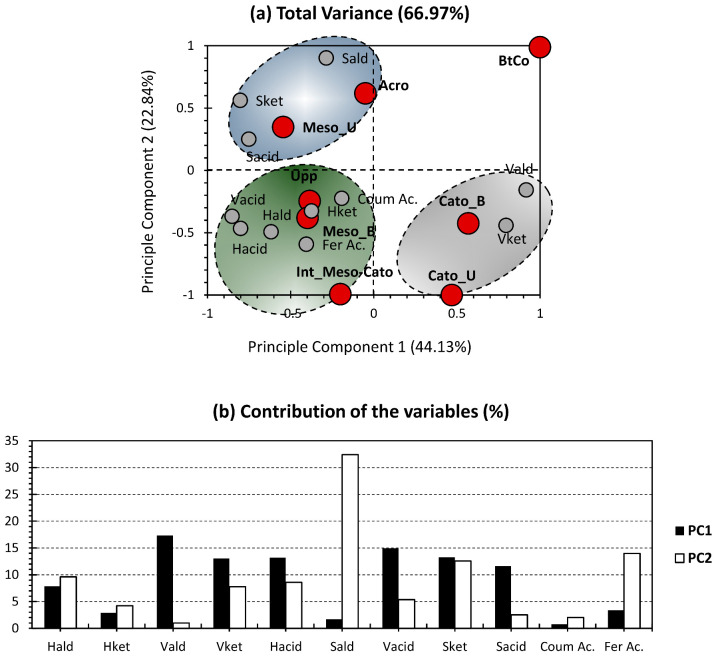
PCA-biplot of the mass fraction for the yielded phenolic sub-units.

Figure 5 presents the PCA bi-plot for the mass fractions of the different sub-units yielded by CuO-NaOH oxidation and shown in Figure 3. The first two PCs accounted for 66.97% of the total variance (44.13% for PC1 and 22.48% for PC2; Figure 5a). PC1 exhibited nearly equally distributed contributions for Vald, Vket, Hacid, Vacid, Sket, and Sacid, ranging from 13% to 17%. For PC_2_, it showed the highest contribution exclusively along Sald, accounting for 32% for this PC’s contribution (Figure 5b). Interestingly, most of the contributors of PC1 are acidic and ketone moieties; this could probably indicate a more oxidized state for depth samples positively influenced and a less oxidized one for samples negatively influenced by this PC. For the trends of the different variables along the PCA bi-plot, S-Compounds showed a certain proximity, with a high positive and slight negative influence of Sald over PC1 and PC2, respectively. For Sacid, it showed a negative trend along PC1, with a slight positive one along PC2. The aforementioned three moieties represent the blue cluster. Unlike the S-compounds, V-compounds were not regrouped together, as a proximity was noticed between Vald and Vket, forming the grey cluster. These two compounds showed a positive influence along PC1, with a mild-to-average influence along PC2. The green cluster gathered the variables of the investigated phenolic sub-units and showed a negative influence along both PCs.

For the individuals, all depth records showed a high dispatchment, relative to each other (Figure 5a). This is acceptable in the sense that the chosen peat samples should be drastically different from each other. This prevents redundancy in the molecular dataset, which makes data interpretation and implementation even harder. The only exception is in the high proximity between Upp and Meso_B. This similarity could be either due to the statistical fallacy of data, or could come from the fact that these depth records are similar in that they present the upper layer of the column. On one hand, the peat core will be emerged with water during the winter, due to the high level of precipitation in the studied region. During this period, the uppermost level (Upp) will be the first and only level in direct contact with atmospheric oxygen. On the other hand, the peat core will be submerged in the summer, due to dry conditions. During this period, the first level on the top of the water column, in direct contact with water and O_2_, is the bottom part of the mesotelm (Meso_B). Interestingly, the bottomhole depth record (BtCo) was excluded from the three investigated clusters. This is due to the peculiar situation of this sample following its interaction with the mineral matrix (Figure 5a).

#### 3.3.2. Application of PCA for Diagenetic Proxies

**Figure 6:**
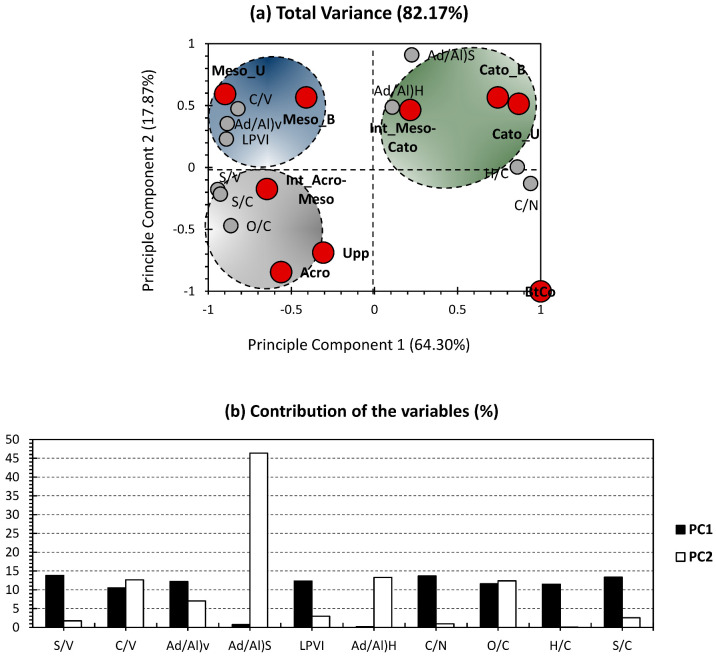
PCA-biplot of phenolic sub-units’ ratios, along with bulk elemental analysis proxies.

Figure 6 presents the PCA bi-plot for the bulk analysis and phenolic sub-units ratios yielded by CuO-NaOH oxidation and presented in Figure 4. The first two PCs accounted for 82.17% of the total variance (64.30% for PC1 and 17.87% for PC2; Figure 6a). The higher variance, compared to the PCA of the mass fractions (Figure 5a), indicates more accordance between the investigated variables and allows to validate the applicability of molecular ratios as indicators of OM source and dynamics along a peat core. PC1 exhibited nearly equally distributed contributions for S/V, C/V, Ad/Al)v, LPVI, C/N, O/C, H/C, and S/C ranging from 10% to 14%. For PC_2_, it showed the highest contribution exclusively along Ad/Al)s, accounting for 46% for this PC’s contribution (Figure 6b). The nearly equal contribution for almost all ratios indicates their relevance to the bulk and molecular characterization of the peat column. The only exception is for Ad/Al)S and Ad/Al)H, as these two showed minor influence at this PC, yet an average-to-high influence at PC2. Since PC1 encompasses higher variance than PC2, the two previously mentioned ratios can be considered as being of low relevance to this particular case study.

With regard to the individuals, better arrangement was noticed for the different depth records. In fact, each of the three ecological layers occupied a quarter of the PCA-biplot, and the last one was exclusively occupied by the bottomhole depth (BtCo; Figure 6a). Similarly to the PCA of Figure 5a, BtCo was discarded from the rest of the individuals, and no variables were positively influenced along its position, on the negative sides of both PCs. As for the acrotelm (Grey Cluster), it is presented here by three samples (Upp, Acro, and Int_Acro-Meso; Figure 6a) and shows a negative influence and both PCs. This cluster was most likely influenced by S/V, S/C, and O/C. With regard to the blue cluster, it gathered around two samples of the mesotelm (Meso_U and Meso_B) and presents a negative and positive influence along PC1 and PC2, respectively. The catotelm (Green Cluster) is shown here by three samples (Int_Meso-Cato, Cato_U, and Cato_B; Figure 6a) and presents a positive influence and both PCs. This cluster was most likely influenced by Ad/Al)s, Ad/Al)H, H/C, and C/N.

#### 3.3.3. PCA & Degradation Parameters

**Figure 7:**
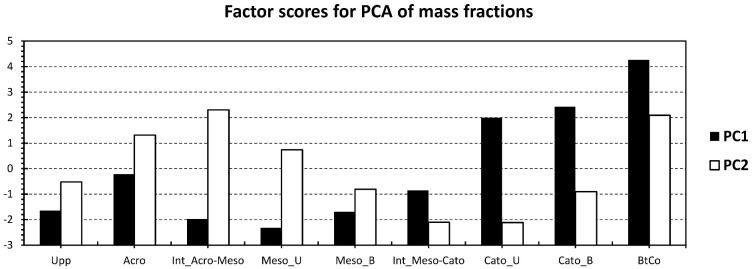
Contribution of the first two principle components obtained from the PCA of Figure 5, along with the depth records of the investigated peat core.

**Figure 8:**
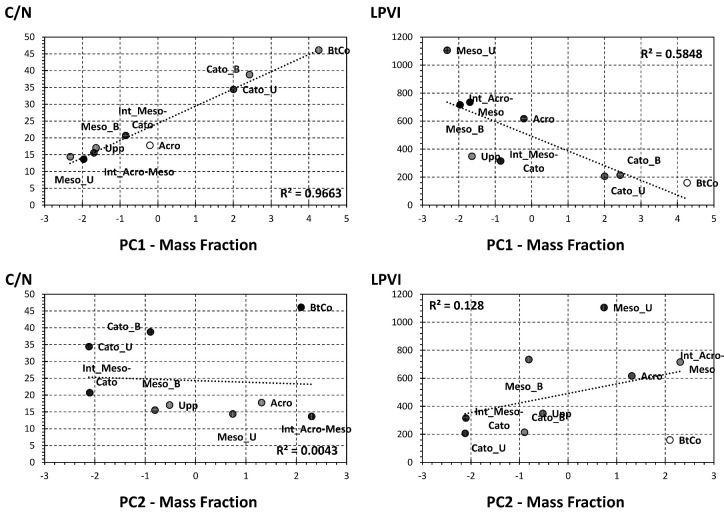
Selected correlation between bulk analysis proxies and the first two principle components yielded by the PCA-biplot of Figure 5.

For the sake of getting new proxies of lignin source vegetation and degradation, we show the depth profile of the first two PCs yielded by PCAs performed on mass fractions (Figure 7). Interestingly, PC1 showed a nearly similar profile to C/N (Table 1), with the lowest inputs in the upper half (acrotelm and mesotelm) and the highest one in the bottom half (catotelm). The fact of having two compartments reveals the “diplotelmic character” of a peatland, where a rapid burial of fresh OM matter occurs in the oxic top half and a conservation of deposited OM in the bottom layer, where anoxic conditions are present [10]. For PC2, it showed a similar trend as source vegetation proxies S/V, C/V, and LPVI (Figure 4), with the highest inputs in the mesotelm layer (Int_Acro-Meso; Figure 7) and at Bottomhole of the column (BtCo; Figure 7). In order to confirm or infirm the aforementioned properties, both PCs were plotted against C/N and LPVI (Figure 8). Interestingly, PC1 and C/N showed a high correlation (96.63%, Figure 8); these trends confirm the capacity to apply PC1 as source vegetation indicator. LPVI showed a higher correlation with PC1 than PC2. This confirms the potential of the application of LPVI to decipher vegetation change, even in a dynamic system such as the peatland.

## 4. Conclusions

In this study, we aimed to seek the efficiency of unsupervised learning for the estimation of lignin degradation along a peat core. The “Principal Component Analysis” (PCA) approach was applied. The benefits of using PCA resides in its capacity to produce new factors that are independent (orthogonal projection) from each other, yet dependent from all of the original factors. Herein, the individuals are nine peat core depth samples, and the variables are the 11 phenolic sub-units yielded by CuO-NaOH oxidation. The separation variables technique is of utmost importance for the analysis of a complex and dynamic system such as peatlands. In this ecosystem, two main phases of OM exists, preservation or degradation. In order to confirm these phases, several proxies were adopted. The preservation of phenolic OM is interpreted by high values of SVC and reflects the occurrence of an old vegetation following peatlands’ deposition over time. For the sake of identifying the sources of this vegetation, S/V and C/V were adopted. For the degradation phase of phenolic OM, Ac/Ad for S- and V-compounds could be used. One shortcoming that could be identified for these proxies is the fact that they could be biased due to the high degradation rate of lignin. This was noticed following the strong decline of the SVC ratio from the uppermost vegetation to the bottom hole of the peat core. Following this trend, and in order to overcome the bias created from the simultaneous degradation and preservation pathways, a more sophisticated ratio for source vegetation indication was adopted: the LPVI. This ratio showed similar trends as the lignin degradation indicators (Ac/Ad), which puts its reliability into question. One PCA was applied for the mass fraction of the different phenolic sub-units and allowed for a separation to be made between the ecological layers and their interfaces. With regard to factors, it allowed for the compilation of two indicators; one for change in vegetation and the other for the degradation of lignin in a peat core along depth. The different results were confirmed by a high correlation along with bulk elemental analysis proxies.

## Figures and Tables

**Table 1:** Details of the nine investigated samples and elemental analysis.

Samples Designation	Description	Depth (cm)	N	C	H	S	O
Upp	Upper vegetation with the underlying soil	4	2.36	40.27	3.99	6.78	36.94
Acro	Acrotelm samples	12	1.45	25.80	3.00	5.56	24.79
Int_Acro-Meso	Interface between acrotelm and mesotelm	24	1.99	27.28	3.46	4.98	23.61
Meso_U	Upper part of mesotelm	32	1.52	22.01	3.03	3.87	18.71
Meso_B	Bottom part of mesotelm	44	1.53	23.81	3.15	2.92	19.81
Int_Meso-Cato	Interface between mesotelm and catotelm	56	1.84	38.23	4.35	4.15	25.18
Cato_U	Upper part of Catotelm	72	1.39	47.76	4.98	2.15	27.71
Cato_B	Bottom part of Catotelm	92	1.02	39.69	4.10	3.24	22.80
BtCo	Bottomhole of the core	100	0.85	39.33	3.71	1.5	27.02

**Table 2:** Lignin phenol vegetation index (LPVI) value ranges obtained for specific plants. Adapted with permission from Ref. [8]. Copyright (2023) with permission from Elsevier.

Plant Types	LPVI Value Ranges		
	Low	Mean	High
Woody Gymnosperms	1	1	1
Non-woody Gymnosperms	12	19	27
Woody Angiosperms	67	181	415
Non-woody Angiosperms	378	1090	2782

## Data Availability

The manuscript has no associated data.
